# A plaque recognition algorithm for coronary OCT images by Dense Atrous Convolution and attention mechanism

**DOI:** 10.1371/journal.pone.0325911

**Published:** 2025-06-10

**Authors:** He Meng, Ran Zhao, Ying Zhang, Bo Zhang, Cheng Zhang, Di Wang, Jinlu Sun

**Affiliations:** 1 Chest hospital, Tianjin University, Tianjin, China; 2 School of Electronics & Information Engineering, Tiangong University, Tianjin, China; 3 Tianjin Key Laboratory of Optoelectronic Detection and System, Tiangong University, Tianjin, China; 4 College of Computer and Information Engineering, Tianjin Normal University, Tianjin, China; Khalifa University, UNITED ARAB EMIRATES

## Abstract

Currently, plaque segmentation in Optical Coherence Tomography (OCT) images of coronary arteries is primarily carried out manually by physicians, and the accuracy of existing automatic segmentation techniques needs further improvement. To furnish efficient and precise decision support, automated detection of plaques in coronary OCT images holds paramount importance. For addressing these challenges, we propose a novel deep learning algorithm featuring Dense Atrous Convolution (DAC) and attention mechanism to realize high-precision segmentation and classification of Coronary artery plaques. Then, a relatively well-established dataset covering 760 original images, expanded to 8,000 using data enhancement. This dataset serves as a significant resource for future research endeavors. The experimental results demonstrate that the *dice* coefficients of calcified, fibrous, and lipid plaques are 0.913, 0.900, and 0.879, respectively, surpassing those generated by five other conventional medical image segmentation networks. These outcomes strongly attest to the effectiveness and superiority of our proposed algorithm in the task of automatic coronary artery plaque segmentation.

## 1 Introduction

Cardiovascular diseases have emerged as a significant peril to human life, necessitating advanced diagnostic approaches [1]. In this context, high-resolution intravascular imaging has emerged as a crucial modality for the diagnosis of such diseases [2]. Computed tomography (CT) is a frequently utilized technique for cardiac vascular imaging. However, CT imaging typically requires more time and provides lower resolution outputs [3]. In contrast, OCT [4] imaging exhibits a relatively faster process and high resolution and contrast capabilities when evaluating the microstructure of the coronary vascular lining. Consequently, OCT imaging has emerged as the latest diagnostic modality for coronary artery plaques [5]. The current reliance on manual assistance from physicians for the interpretation of OCT images is time-consuming and labor-intensive. Furthermore, there exists subjective variability in the interpretation of OCT image results among different practitioners. Consequently, there exists an imperative requirement to investigate a high-precision automatic plaque segmentation algorithm founded on coronary OCT images. The deployment of such an algorithm would significantly enhance clinical diagnosis by delivering a more objective and efficient approach.

In recent years, significant advancements have been made in automatic medical image segmentation techniques [6]. Notably, numerous scholars have directed their efforts towards the classification and segmentation of plaques in coronary OCT images [7]. Nils et al. [8] employed a deep learning algorithm, deviating from traditional machine learning methods, to construct a Convolutional Neural Network (CNN) by combining ResNet50-V2 [9] and DenseNet-121 [10]. The primary objective was to determine the presence of plaques in OCT images and classify them into categories including ‘no plaque’, ‘calcified plaque’, and ‘fibrous/lipoidal plaques’. However, the study did not address the segmentation task and solely focused on classification. Moreover, the dataset used for training and evaluation was relatively limited, which posed a potential risk of overfitting. Lee et al. [11] conducted automated segmentation of lipid plaques and calcified plaques in intravascular grayscale OCT images using the SegNet [12] and Deeplab v3+ [13], achieving *dice* coefficients of 0.801 and 0.734, respectively. Gharaibeh et al. [14] leveraged the SegNet for pre-training the vessel wall and calcified plaque segmentation task. They subsequently utilized Conditional Random Field (CRF) [15] to eliminate noise from the segmentation results, attaining a *dice* coefficient of 0.76. However, both studies [11] and [14] only focused on segmenting one to two types of plaques, and the segmentation accuracy achieved was not optimal. This limitation may result in insufficient analysis of the features and diversity present in the entire dataset, potentially diminishing the accuracy and comprehensiveness of the overall classification. He et al. [16] introduced a ResNet-3D network, which combines a pre-initialized ResNet-50 with a 3D convolutional filter to perform segmentation and classification of calcified plaques in OCT pullback images. Ren et al. [17] employed dense blocks [10] built upon the SegNet architecture to achieve pixel-level segmentation of plaques in OCT images. The segmentation effectiveness of the model was evaluated using ten-fold cross-validation. The incorporation of the ResNet-3D in literature [16] and the utilization of dense blocks in literature [17] enhance the segmentation accuracy. However, this improvement comes at the cost of increased network depth and computational parameters. As a result, processing large-scale data with such models requires higher graphics memory and may lead to less efficient training.

Deep learning algorithms developed by researchers for automated segmentation of plaques in coronary OCT images have made remarkable advancements. However, they still exhibit certain limitations:

(1) Due to the high variability in the location, size, and volume of coronary plaques, traditional CNN typically rely on ordinary convolutional kernels for feature extraction, which employ a static computation model [18]. This approach has limited learning capacity and feature extraction capabilities. Specifically, it struggles to capture fine-grained details, particularly key features such as plaque locations and boundaries, which are often not effectively highlighted [19]. Consequently, many crucial details may be overlooked.
(2) Given the complex morphology of coronary plaques, stacked ordinary convolutions are commonly used to enhance the learning capability of CNN [20]. This multi-layer convolutional architecture allows the network to capture more detailed information about the plaque, thereby facilitating high-precision segmentation of plaques with complex structures. However, as the number of layers increases, the computational complexity rises exponentially, leading to longer processing times and reduced computational efficiency due to the higher demands on arithmetic and inference [21].
(3) Due to the limited availability of publicly accessible datasets and the challenges in obtaining an adequate number of training samples, researchers have focused their studies primarily on one or two types of coronary plaques [22]. This approach may lead to overfitting issues during the training process and a lack of understanding regarding potential features associated with other plaque types.

To address the aforementioned issues, we propose a convolutional neural network that combines DAC with attention mechanisms to achieve high-precision automated identification and segmentation of three types of coronary plaques: calcified, lipid, and fibrous. The primary contributions of the work are as follows:

(1) By incorporating the Convolutional Block Attention Module (CBAM) attention mechanism, the limitations of conventional convolutional networks, such as insufficient learning capacity and constrained feature extraction ability, are effectively addressed. The dual-attention mechanism, comprising both channel attention and spatial attention modules, enables the model to dynamically focus on key features. Different plaque types typically exhibit distinct feature representations across various channels. The channel attention module facilitates the identification of channels that contain crucial features relevant to the specific plaque type, thus enhancing the model’s ability to focus on the most informative aspects of the data. For instance, normal tissue and lesion regions may exhibit significant differences in specific channels, and the channel attention mechanism can automatically amplify or suppress the weights of these relevant channels, thereby enhancing the model’s focus on important features. Spatial attention operates by emphasizing the spatial distribution of the input feature map, particularly identifying which localized regions within the image are critical for plaque recognition. This enables the model to focus on both the most informative channels and the most relevant spatial areas, improving overall feature extraction and recognition accuracy.
(2) The introduction of the DAC module, which utilizes convolutional kernels with varying dilation rates, effectively expands the receptive field and enhances global sensing capability without increasing the number of parameters. This approach allows for more accurate capture of plaque location and edge information. Traditional convolution methods expand the receptive field by either increasing the kernel size or stacking additional layers, which results in a quadratic increase in computational complexity. In contrast, dilated convolution achieves the equivalent receptive field of larger kernels (e.g., 5 × 5 or larger) by adjusting the sampling spacing of the convolution kernel, while maintaining the computational efficiency of smaller 3 × 3 kernels. Taking a plaque target larger than 50 pixels in an OCT image as an example, traditional methods require at least 5 × 5 standard convolution kernels to match the target size. In contrast, the DAC module can achieve the same receptive field with only 3 × 3 dilated convolutions (d = 2). The DAC module effectively captures the global features of large-sized lesion areas, while circumventing the computational burden associated with traditional approaches that expand the receptive field by increasing the convolution kernel size or the network depth. By doing so, the DAC module offers a more efficient solution for capturing global features without the added computational cost.
(3) A relatively comprehensive dataset has been constructed to evaluate the segmentation performance of three types of plaques: calcified, fibrous, and lipid plaques. This dataset consists of 8000 images for each type of plaque, obtained from 60 patients. To some extent, this dataset addresses the shortage of data in the current research field, providing a foundation for simultaneous studies of the three plaque types and reducing the risk of overfitting due to the limited dataset size.
(4) Visualization of the prediction results. The OCT images to be predicted were simultaneously input into three models with the ability to identify specific plaques. The output predictions were subjected to contour detection using an edge detection algorithm, and the calcified, fibrous, and lipid plaques were differentiated and displayed using red, blue, and yellow colors, respectively. Accurate classification of different plaques allows for a more in-depth understanding of the lesion’s characteristics, thus providing more comprehensive and personalized guidance for clinical diagnosis and treatment.

## 2. Materials and methods

The current research was granted approval by the Ethics Committee of Tianjin Chest Hospital (IRB-SOP-016(F)-001–03), and obtained verbal consent. The study sample consisted of patients admitted to the hospital from January 2024 to June 2024, from whom coronary optical coherence tomography (OCT) imaging data were collected. Data from admitted patients were accessed on September 6, 2024, for study purposes. Authors had not access to information that could identify individual participants during or after data collection.

### 2.1 Dataset

#### 2.1.1 Data acquisition.

Due to the limited size of the publicly available coronary OCT image dataset, which impacts the efficacy of network training, we constructed a custom dataset tailored to meet the experimental criteria. The images were captured using an optical interferometric tomography system commonly used in clinical settings. This system comprises an imaging catheter, a catheter retraction mechanism, and an OCT host platform. The catheter was navigated along the guidewire to reach the lesion site and subsequently retracted for imaging at a speed of 20 mm/s within vessels measuring 40–60 mm in length. The catheter was navigated along the guidewire to reach the lesion site and subsequently retracted for imaging at a speed of 20 mm/s within vessels measuring 40–60 mm in length.

**Fig 1:**
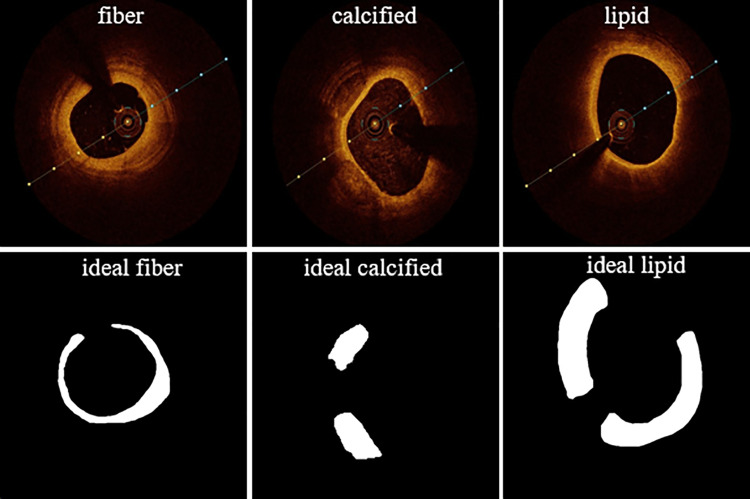
Original and ideally segmented images of the three plaques. From left to right, the OCT images of fibrous plaques, calcified plaques, and lipid plaques.

The study sample comprised 60 patients, from whom in vivo coronary artery OCT imaging data were collected. This cohort consisted of 30 males and 30 females, with a mean age of 64 years. Out of the 60 segments of complete pullback information obtained, image frames exhibiting poor quality, characterized by indistinct lumen boundaries, blood residue within the lumen, and imaging artifacts, were omitted. Subsequently, a total of 760 raw OCT images were selected for both model training and testing purposes. Each image was labeled by two experienced physicians, and a third expert reviewed the labeling results, offering feedback and corrections where necessary. This meticulous process ensures the professionalism and accuracy of the labeling work. The dataset comprises three distinct types of plaques: calcification, fibrous, and lipid, with an illustrative example of the labeling results for each plaque type depicted in Fig 1.

#### 2.1.2 Data preprocessing.

Training convolutional neural networks requires a large volume of images, but manual labeling is both costly and time-consuming, and medical image data is often difficult to obtain. To address this, data augmentation techniques are commonly employed to expand the dataset [23]. Standard data augmentation methods include rotation, flipping, luminance transformation, cropping, scaling, and noise addition. However, for OCT images, cropping and scaling may lead to the loss or distortion of key morphological information of the plaques, potentially affecting the model’s recognition accuracy. In contrast, rotation and flipping enhance data diversity while preserving the integrity of image features. Additionally, noise addition improves the model’s robustness, enabling it to better withstand noise interference. In this paper, data augmentation techniques such as rotation, flipping, and noise addition are employed to effectively expand the training dataset while ensuring the preservation of data quality. This approach provides a solid data foundation for the accurate recognition by the subsequent model. Ultimately, 680 original images containing plaques and 80 original images without plaques are augmented to generate 8,000 images of calcified plaques, 8,000 images of lipid plaques, and 8,000 images of fiber plaques. Through transformations and expansions, the model becomes less reliant on specific training samples and is able to learn the invariant and generalizable features inherent to each type of plaque.

### 2.2 Experimental environment

Training was performed using Windows 10 operating system on a CPU model Intel(R) Xeon(R) W-2245 and a graphics card NVIDIA GeForce RTX 4090 24GB, with the model loaded in Python 3.8, Pytorch 2.0.1 framework.

### 2.3 Experimental procedure

**Fig 2:**
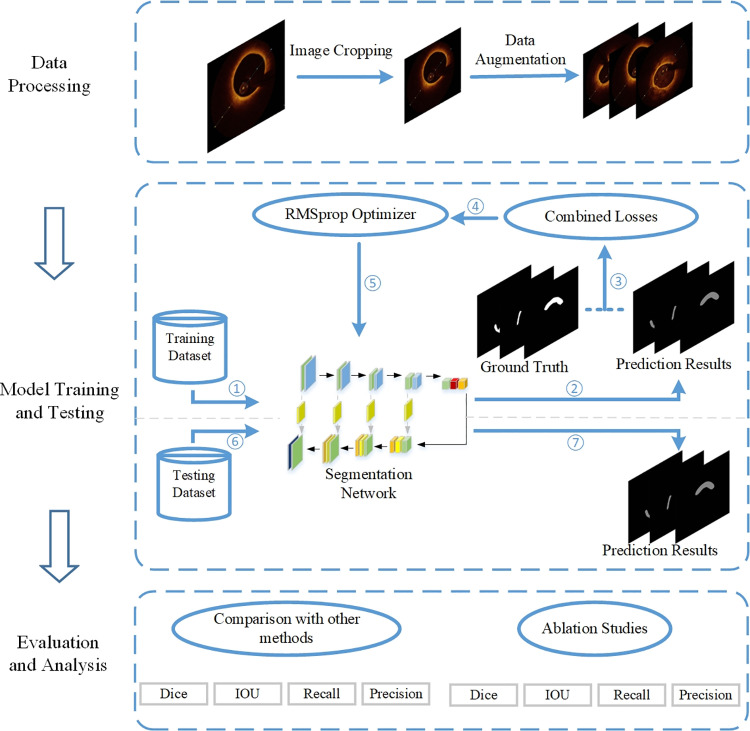
Workflow of this paper.

The workflow delineated in Fig 2 encompasses three primary segments. The first part focuses on data processing, primarily involving operations such as OCT image cropping and data augmentation. The second part involves model training and testing. The datasets containing three types of plaques are input into the network respectively. Parameters are adjusted by learning the features from the samples in the training set. After training is completed, three models capable of recognizing specific plaques are obtained. Subsequently, the test set is used to verify the training effect of the network. The third part is the evaluation and analysis, where the model is evaluated using four metrics for comparative and ablation experiments.

### 2.4 Network architecture

**Fig 3:**
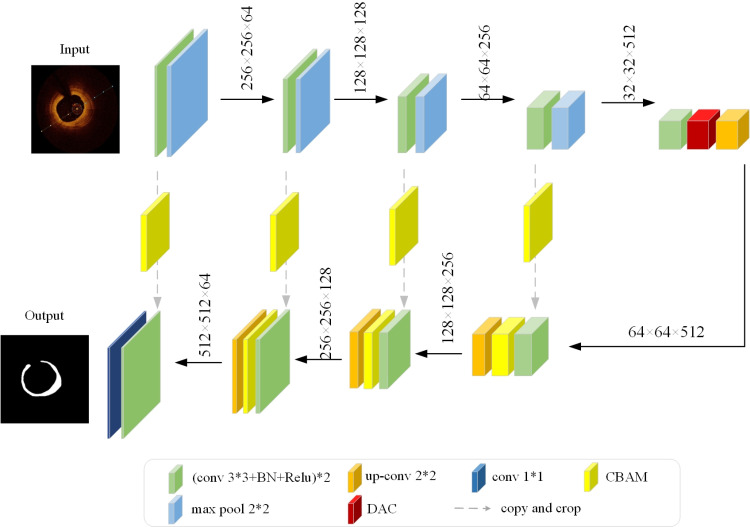
Improved network used in this paper.

Coronary OCT images encompass a plethora of low-level semantic features, encompassing attributes like location and texture, which are as pivotal as the implicit high-level semantic features [24]. However, the pooling operation risks compromising the retention of intricate details. To mitigate this issue and enhance the accuracy of plaque segmentation, a U-Net architecture incorporating skip connections is adopted as the foundational network [25]. The Skip Connection structure within U-Net facilitates the direct transmission of information across distinct layers through the establishment of skip connections linking shallow features with deeper ones [26]. This mechanism aids in the sustained preservation and utilization of low-level features during the extraction of deeper features. Furthermore, in the task of plaque segmentation, it is necessary to enhance the network to accommodate different modal features. Therefore, a simple and lightweight U-Net network structure exhibits greater scalability. Nonetheless, the conventional U-Net architecture tends to be susceptible to issues arising from the imbalanced distribution of positive and negative samples, often leading to the neglect of regions with limited representation of the target [27]. To mitigate these challenges, this paper introduces a segmentation algorithm that integrates DAC and attention mechanisms to enhance U-Net. The proposed network structure is illustrated in Fig 3.

In the encoding path, located in the upper segment of the network, a 3 × 3 convolutional kernel is iteratively employed for feature extraction, adhering to the established structure commonly observed in convolutional neural networks [28]. The inclusion of a Batch Normalization (BN) [29] layer following each convolutional unit is instrumental in centering the data around a mean of 0 and a variance of 1. This normalization process aids in limiting the data’s range of variation, mitigating the impact of large data fluctuations on the training process. Consequently, it enhances the stability and convergence of the model. After normalization, the Rectified Linear Unit (ReLU) [30] function is employed as the activation function due to its computational efficiency and its ability to alleviate the gradient vanishing problem. The function is expressed as follows:


$$\begin{array}{c} \begin{array}{c} f(x)=max(0,x) \end{array} \end{array}$$ (1)

Given the inherently linear nature of operations within the convolution process, their expressive capacity is constrained. The introduction of an activation function serves to nonlinearly map the output of the convolution, thereby empowering the network to tackle more intricate segmentation tasks while augmenting the model’s expressive capabilities. To obtain global features, down-sampling is performed using the max-pooling function to gradually reduce the feature map size [31]. Before each down-sampling operation, the CBAM is incorporated. It processes the features extracted from the preceding convolutional layer through a channel attention mechanism, which weights the results, followed by a spatial attention mechanism. This approach directs the model’s focus towards the location-specific features of plaques within the coronary arteries. For instance, lipid plaques are frequently situated in the outer membrane of coronary arteries, while fibrous plaques tend to be located in the intima of the coronary artery. This refinement process enhances the original features. Following each down-sampling operation, the dimensions of the image are decreased to one-fourth of its initial size, while simultaneously doubling the number of channels. Upon four iterations of the down-sampling operation, the image size dwindles from 512 × 512–32 × 32, accompanied by an augmentation in the channel dimensions from 3 to 1024.

In the lower decoding pathway, feature maps are up-sampled using deconvolution [32], a process that remaps the feature maps from the low-resolution space of the encoding pathway back to the resolution of the original input image for pixel-level prediction. Each up-sampling component comprises two 3 × 3 convolutions along with a ReLU function. Deep convolution encounters challenges such as information loss and resolution degradation during up-sampling. To address this, a DAC module is incorporated before the initial up-sampling step. This module conducts convolutional feature extraction across various scales by stacking multiple Atrous Convolution, thereby enhancing contextual information acquisition. Before the second through fourth up-sampling operations, the CBAM attention mechanism was once more employed to refine the features acquired both in terms of channel and spatial dimensions. This application aimed to mitigate interference originating from the background, thereby enhancing the accuracy of segmentation outcomes. Subsequently, the number of output channels is modified to 2 utilizing 1 × 1 convolutional operations to align with the count of target categories. Following each up-sampling step, the image dimensions are increased by a factor of four compared to the input, while the channel count is halved. Through four iterations of the up-sampling process, the image dimensions are reinstated to 512 × 512.

Through the implementation of skip connections between the encoding and decoding stages, shallow features extracted during encoding and deep features generated subsequent to up-sampling in the decoding phase are amalgamated and fused along the channel dimension. This integration enhances contextual information provision and mitigates information loss, thereby enriching global features and refining segmentation outcomes for improved accuracy.

### 2.5 DAC

Within the network encoding phase, convolution and pooling operations are executed on the image, resulting in the loss of crucial information, subsequently impacting the ultimate segmentation efficacy. To capture a broader scope of contextual details, the convolution kernel size is typically expanded to augment the receptive field. However, this adjustment invariably results in an exponential surge in parameter count. Atrous Convolution [33] employs an expansion rate of the kernel minus 1 to sample feature maps at intervals. By utilizing Atrous Convolution instead of standard convolution, it becomes possible to expand the receptive field without a concomitant increase in parameter count. The receptive field, denoted as *F*, is calculated as follows:


$$\begin{array}{c} F=(r-1)\times (k-1)+k \end{array}$$ (2)

Where represents the dilation rate of Atrous Convolution, and *k* denotes the size of the convolution kernel. When the dilation rate is 1, it corresponds to standard convolution.

**Fig 4:**
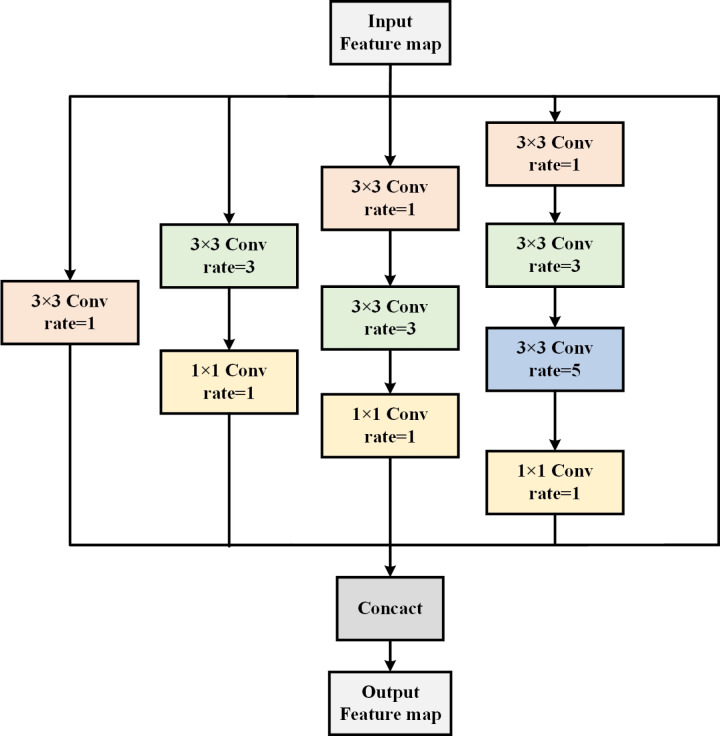
DAC module.

To achieve a more adaptable receptive field, the Inception [34] model is integrated Atrous Convolution, resulting in a Atrous Convolution module with varied dilation rates across its four cascading branches, denoted as the DAC module. As depicted in Fig 4, the convolutional kernels incorporating voids are arranged in a cascaded fashion, facilitating efficient expansion of both depth and width in the network. The number of voids convolved on each branch progressively increases, resulting in receptive field sizes of 3, 7, 9, and 19. Each branch undergoes final linear activation via a 1 × 1 convolution, facilitating down-sampling of the feature map to mitigate computational overhead. Through the utilization of convolutional kernels with varying expansion rates, the network effectively captures features spanning multiple scales, thereby bolstering its capability to discern patches of diverse sizes and configurations. The incorporation of feature information extracted from the four branches into the original feature map, akin to the residual connections observed in ResNet, affords each layer access to the semantic details from its preceding counterpart. This addresses the issue of gradient vanishing during training, thereby fostering deeper optimization of the network architecture.

The introduction of the DAC module between the encoding and decoding pathways of the network addresses the challenge of feature information loss during down-sampling for feature extraction. Through its strategic insertion of nulls, this module effectively reduces the convolutional computation parameters, leading to lower consumption of computational resources while achieving a broader receptive field. Importantly, this approach avoids image resolution compression, enabling the capture of micro features such as fine edge textures in OCT images.

### 2.6 CBAM attention mechanism

In the context of plaque segmentation, conventional algorithms face challenges in dealing with physical attributes such as the small size of coronary artery plaques, their unpredictable locations, and the intricate textures of the plaques. However, by integrating the attention mechanism, these limitations can be mitigated. The attention mechanism effectively suppresses background features present in OCT images, accentuating the plaque information and bolstering the network’s capability to express features distinctly. In the task of OCT image patch segmentation, both spatial and channel information within the feature map play pivotal roles, encompassing aspects like patch location, texture, and luminosity, among others. To harness this comprehensive information effectively, a fusion of spatial attention and channel attention mechanisms is employed. This combined approach is tailored to address the intricate characteristics of image patches and meet the segmentation demands effectively.

**Fig 5:**
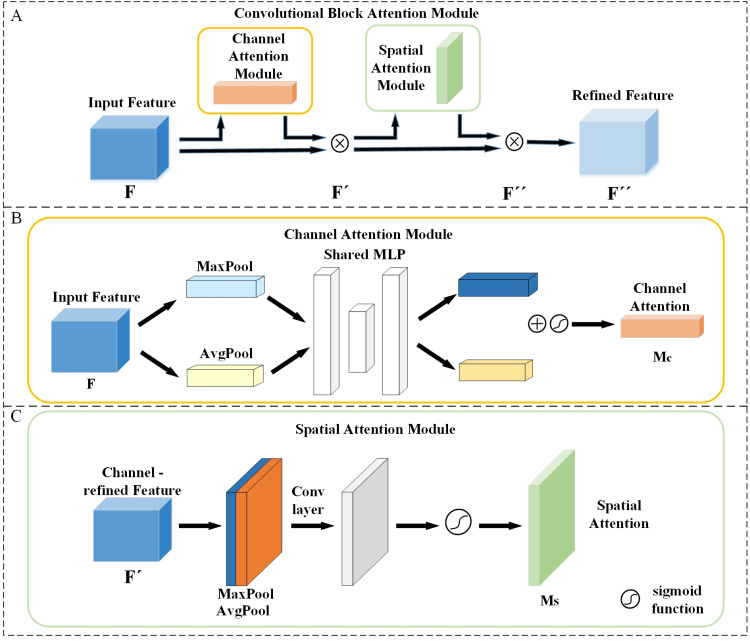
CBAM attention mechanism.

The CBAM [35], excels in capturing both spatial and channel dependencies within feature maps. Illustrated in Fig 5A, this mechanism operates by multiplying the feature information extracted from both channel and spatial aspects with the original features at the element level. This process results in adaptively refined features, thereby enhancing the pixel-level classification accuracy during image recognition tasks.

In the context of a feature map $F\;\in \;R^{C\times H\times W}$, where C represents the number of channels, H denotes the width, and W signifies the length of the feature map, the initial step involves the application of the channel attention module. The structure of this module is depicted in Fig 5B. The input feature maps undergo processing through two parallel layers: maximum pooling and average pooling. Subsequently, the resulting outputs are forwarded through a shared Multilayer Perceptron (MLP). The outputs from this MLP are then summed element-wise. Finally, the resultant sum is activated using a sigmoid function, yielding the channel attention feature $M_{c}\;\in {\;R}^{C\times 1\times 1}$. Channel attention mechanisms in OCT image analysis enable the differentiation of features across channels, facilitating the adaptive weighting of channels based on their feature importance. This allows the model to prioritize plaque features, which have a more significant influence on segmentation outcomes. Concurrently, it suppresses irrelevant background features, thereby reducing interference and enhancing the overall segmentation performance.

The original feature map *F* is multiplied by $M_{c}$ to obtain the feature map $F^{\prime }$, which contains plaque channel information. Subsequently, the feature map $F^{\prime }$ is input into the spatial attention module, the structure of which is illustrated in Fig 5C. After passing through the maximum pooling layer and the average pooling layer, the features are concatenated and integrated. Following a convolutional operation and activation by the sigmoid function, the original feature map undergoes a transformation into an attention map, denoted as the spatial attention feature $M_{s}\;\in {\;R}^{1\times H\times W}$. By element-wise multiplication of $F^{\prime }$ with the corresponding elements of $M_{s}$, the optimized feature map (*F”*) is derived, encapsulating both plaque spatial and channel information. This process can be succinctly described as follows:


$$\begin{array}{c} F^{\prime }=M_{c}(F)⊗F \end{array}$$ (3)

$$\begin{array}{c} F^{\prime \prime }=M_{s}\left(F^{\prime }\right)⊗F^{\prime } \end{array}$$ (4)

Spatial attention is employed to compute the significance of individual pixels within the OCT image based on their spatial coordinates, thereby facilitating a more precise capture of the spatial structure information pertaining to the plaque. Through the generation of a weight map designed to accentuate critical regions within the image, the model can more precisely identify pivotal points, consequently enhancing segmentation accuracy.

### 2.7 Combined losses

In the realm of image segmentation, the cross-entropy loss function has gained recognition as an efficacious metric [36]. Employing the binary cross-entropy loss function enables the evaluation of the dissimilarity between predicted outcomes and ground truth labels, thereby augmenting the model’s capacity to discern intricate regions and refine segmentation accuracy at boundaries through penalizing inaccurate predictions. Given the inherent challenge of non-uniform sample distribution within OCT images, characterized by a smaller proportion of plaque area juxtaposed with a larger background area, the binary cross-entropy loss function exhibits heightened sensitivity to this issue [37]. In contrast, the *dice* loss function provides a more equitable treatment of structures across varied sizes, prioritizing the maximization of overlap between predicted and ground truth results. Consequently, this paper adopts a combined loss function merging the binary cross-entropy loss function $L_{BCE}$ with the *dice* loss function $L_{Dice}$ as the ultimate loss criterion. The incorporation of the *dice* loss function serves to refine the model’s attention towards patches, augment sensitivity to minute target areas, and promptly mitigate biases stemming from substantial background interference.

The specific formulae for the combined loss function are as follows:


$$\begin{array}{c} Loss=L_{BCE}+L_{dice} \end{array}$$ (5)

The binary cross-entropy loss function is calculated as:


$$\begin{array}{c} \begin{array}{c} L_{BCE}=-\frac{1}{N}\sum_{i=1}^{N}\left[y_{i}\ln\left(p_{i}\right)+\left(1-y_{i}\right)\ln\left(1-p_{i}\right)\right] \end{array} \end{array}$$ (6)

Where *N* is the sum of the number of pixel points in the input image; $y_{i}$ is the label to which the image belongs; and $p_{i}$ is the probability that each pixel point is predicted.

The *dice* loss function is calculated as:


$$\begin{array}{c} \begin{array}{c} L_{dice}=1-\frac{2|X\cap Y|+smooth}{|X|+|Y|+smooth} \end{array} \end{array}$$ (7)

Here, |X| represents the pixel count of the ground truth, |Y| denotes the pixel count of the predicted output, and a smoothing parameter is typically introduced to avoid division by zero in the denominator.

The amalgamation of the cross-entropy loss with the *dice* loss function not only enhances the model’s capacity to discern diminutive sample regions but also facilitates model optimization, thereby expediting convergence. Furthermore, it mitigates the risk of overfitting to some extent, as it necessitates simultaneous fulfillment of the optimization requisites of both the binary cross-entropy loss function and the *dice* loss function.

## 3. Results

### 3.1 Evaluation indicators

The model employs *dice* coefficient, *precision*, *recall*, and *intersection over union ratio (IOU)* as evaluation metrics. In medical image segmentation, *dice* is widely regarded as a robust metric, indicating the level of similarity between the predicted and actual results [38]. *precision* and *recall* serve to evaluate the classification accuracy of positive and negative samples within segmentation results. They are calculated using the following formulas: True Positives (TP) represents cases where both predicted and actual data are true; True Negatives (TN) represents cases where both predicted and actual data are false; False Positives (FP) represents cases where predicted data are true but actual data are false; and False Negatives (FN) represents cases where predicted data are false but actual data are true.

*dice* coefficient is defined as:


$$\begin{array}{c} \begin{array}{c} dice=\frac{2\times TP}{\left(TP+FN)+(TP+FP\right)} \end{array} \end{array}$$ (8)

*precision* is defined as:


$$\begin{array}{c} \begin{array}{c} precision=\frac{TP}{TP+FP} \end{array} \end{array}$$ (9)

*recall* is defined as:


$$\begin{array}{c} \begin{array}{c} recall=\frac{TP}{TP+FN} \end{array} \end{array}$$ (10)

*IOU* is defined as:


$$\begin{array}{c} \begin{array}{c} IOU=\frac{TP}{FP+TP+FN} \end{array} \end{array}$$ (11)

The *dice* coefficient assesses similarity by computing the ratio of overlap between predicted results and ground truth labels to their sum. It demonstrates heightened sensitivity in evaluating small targets and effectively captures pixel-level accuracy in segmentation outcomes. In contrast, *IOU* evaluates overlap between predicted results and ground truth labels by determining the ratio of their intersection to their union. Consequently, these two metrics collectively offer a comprehensive evaluation of both coverage and localization accuracy in OCT plaques segmentation results. *Precision* indicates the proportion of samples predicted as plaques that are correct; the higher the value, the more effectively the model avoids misclassifying the background as plaques. *recall* indicates the extent to which the model is able to cover the real plaques, i.e., the model’s detection rate of plaques. A higher *recall* suggests the model can effectively capture plaque samples and minimize misclassification as background.

### 3.2 Experimental results

The experiments were conducted using three types of datasets: calcified plaque, fibrous plaque, and lipid plaque. Ten-fold cross-validation is used as a more reliable validation strategy. With ten-fold cross-validation, the dataset is divided into 10 subsets and one subset is rotated as the test set and the rest as the training set in each iteration to obtain a more comprehensive and stable performance evaluation. This approach effectively reduces the chance bias due to a single data division and improves the statistical reliability of the results. Five models renowned for their excellence in image segmentation were chosen for comparative experiments with the model proposed in this paper, aiming to evaluate its segmentation and recognition performance. Each category of plaques underwent training using U-Net [25], SegNet [12], U-Net++ [20], CENet [39], and Attention U-Net [40], respectively. All models were trained using identical dataset formatting, data augmentation techniques, and training parameters: an initial learning rate of 0.0001, 120 epochs, a batch size of 32, Root Mean Square Propagation (RMSprop) optimization.

The plaque prediction results are displayed in Table 1, Table 2 and Table 3, with the optimal outcomes for each metric highlighted in bold. Among the three types of coronary artery plaques: calcified, fibrous, and lipid, calcified plaques exhibit distinctive features in OCT images, characterized by low reflectivity, low attenuation, and clear boundary contours, leading to superior training efficacy. Conversely, fibrous and lipid plaques lack prominent boundary characteristics in the images, resulting in overall lower training performance compared to calcified plaques.

**Table 1:** Comparison of predictive performance of models for calcified plaque.

Networks	*precision*	*recall*	*IOU*	*dice*
U-Net	0.922	0.916	0.831	0.904
U-Net++	**0.927**	0.905	0.842	0.908
SegNet	0.905	0.887	0.790	0.878
CENet	0.920	0.854	0.783	0.863
Attention U-Net	0.724	0.902	0.662	0.782
Our method	0.922	**0.924**	**0.842**	**0.912**

**Table 2:** Comparison of predictive performance of models for fibrous plaque.

Networks	*precision*	*recall*	*IOU*	*dice*
U-Net	0.896	0.836	0.773	0.850
U-Net++	0.890	0.811	0.721	0.829
SegNet	0.895	0.882	0.779	0.870
CENet	0.826	0.886	0.724	0.833
Attention U-Net	0.716	**0.952**	0.676	0.800
Our method	**0.921**	0.904	**0.821**	**0.898**

**Table 3:** Comparison of predictive performance of models for lipid plaque.

Networks	*precision*	*recall*	*IOU*	*dice*
U-Net	0.801	0.867	0.700	0.804
U-Net++	0.836	0.901	0.729	0.835
SegNet	0.658	0.635	0.611	0.608
CENet	0.772	0.762	0.590	0.726
Attention U-Net	0.791	0.925	0.719	0.828
Our method	**0.859**	**0.938**	**0.794**	**0.877**

The novel structural network proposed in this paper is compared against several classical medical image segmentation networks. While the segmentation accuracy of the U-Net++ surpasses that of the other benchmark networks, its model memory consumption reaches 8998.73 MB, significantly higher than the model presented in this paper, which occupies 5903.45 MB, thereby resulting in reduced training efficiency. Although the memory footprint of CENet training is lower at 1911.32 MB, its segmentation performance on coronary OCT images is inferior, with a *dice* coefficient of only 0.7214, rendering it unsuitable for such image segmentation tasks. SegNet represents a novel approach to semantic segmentation, offering improved training speed compared to the network presented in this paper. However, its segmentation accuracy falls short. Despite the inclusion of an attention mechanism in Attention U-Net, the CBAM attention mechanism utilized in this study combines feature optimization for OCT images across both channel and spatial dimensions, yielding superior results compared to the attention gating mechanism in Attention U-Net.

The *dice* coefficient, *IOU*, *recall*, and *precision* of calcified, fibrous, and lipid plaques obtained during the training of the model in this paper are presented in the table above. Among these metrics, both the *dice* coefficient and *IOU* outperform those of the other five comparative models. While *recall* and *precision* do not reach optimal levels, they still surpass those of the original U-Net network. These results confirm that the model proposed in this paper effectively enhances segmentation accuracy.

**Fig 6:**
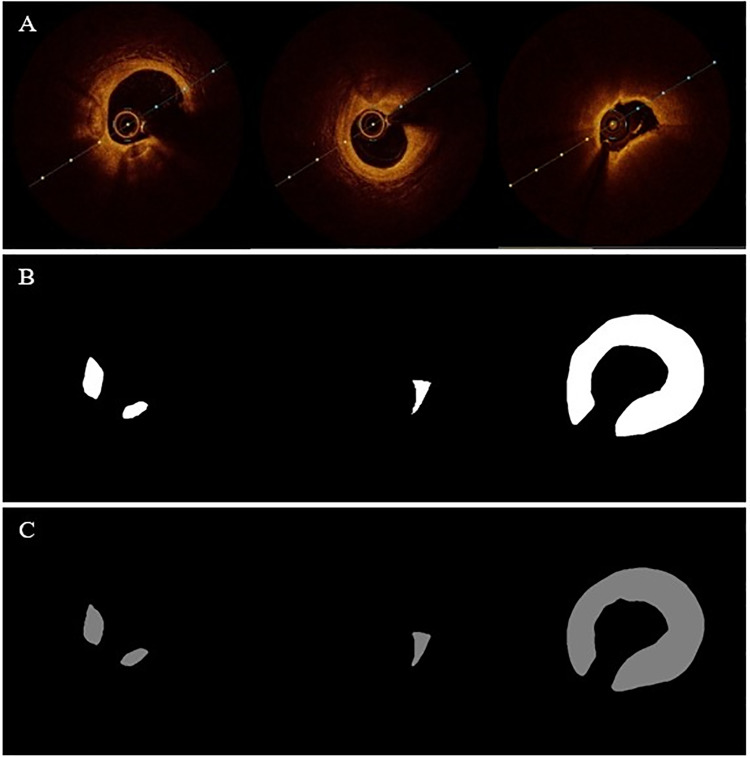
Comparison images of ideal segmentation results and predicted results for three types of plaques. (A) Original OCT image of calcified, fibrous and lipid plaque. (B) Ideal segmentation image of calcified, fibrous and lipid plaque. (C) Predicted segmentation image of calcified, fibrous and lipid plaque.

Table 4 presents a comparative evaluation of the model proposed in this paper with other models proposed in the literature, focusing on the *dice* coefficient and *recall* metrics. The findings demonstrate that our model surpasses those presented in other studies in both metrics. Additionally, the comparison of plaque prediction results with ideal segmentation results is depicted in Fig 6. Fig 6B illustrates the ideal segmentation image, while Fig 6C showcases the plaque images predicted by our model, revealing a high degree of consistency between the prediction outcomes and the ideal segmentation results.

**Table 4:** Comparison of predictive performance of related studies.

Literature	Methods	Database	Results	
[11] 2019	SegNet	4892 images	lipid plaque	*dice* 0.801, *recall* 0.874
			calcified plaque	*dice* 0.734, *recall* 0.851
[14] 2019	SegNet + CRF	2640 images	calcified plaque	*dice* 0.76, r*ecall* 0.85
[16] 2020	ResNet-3D	4860 images	calcified plaque	*dice* 0.823
[17] 2023	SegNet + dense blocks	490 images generated a total of 22, 210 square patches	calcified plaque	*recall* 0.918
			fibrous plaque	*recall* 0.928
			lipid plaque	*recall* 0.917
Our method	U-Net +DAC+CBAM	8000 images	calcified plaque	*dice* 0.912 *recall* 0.924
		8000 images	fibrous plaque	*dice* 0.898 *recall* 0.904
		8000 images	lipid plaque	*dice* 0.877 *recall* 0.938

### 3.3 Visualization of patch prediction results

**Fig 7:**
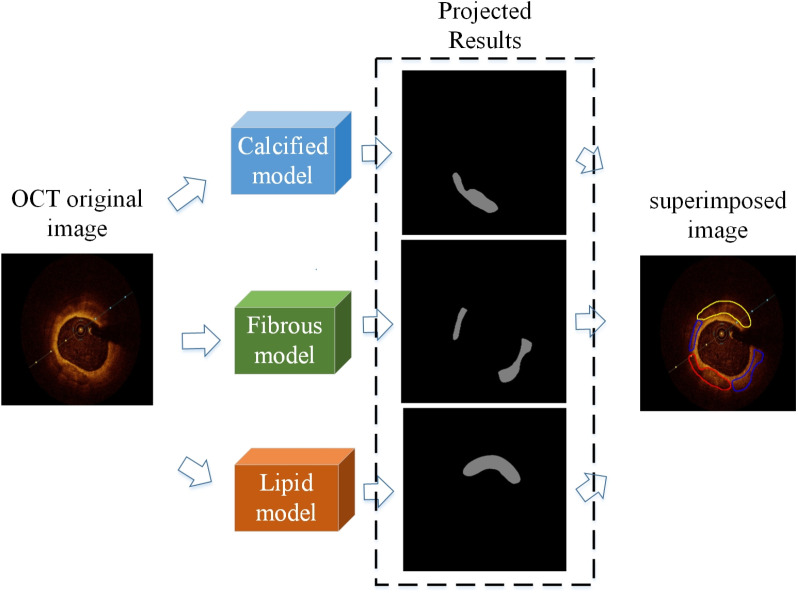
OCT images containing three plaques predicted by different models.

A randomly selected OCT image containing all three types of plaques is depicted in Fig 7. The original image is simultaneously inputted into three trained models, each responsible for segmenting calcified, fibrous, and lipid plaques within the image, respectively. In cases where the specified type of plaque is present, each model will output corresponding markers. Conversely, if the specified plaque type is absent, an all-black image will be generated as output.

**Fig 8:**
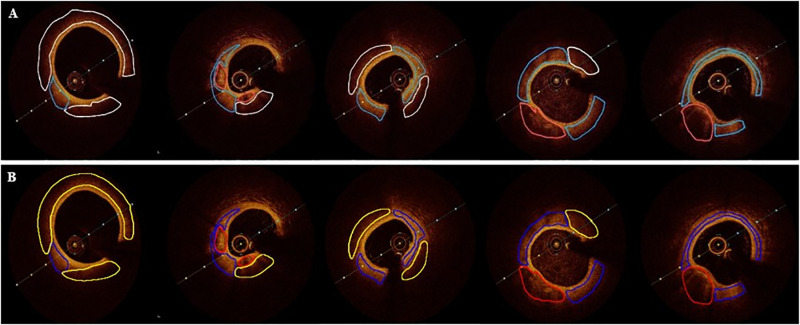
Visualization results on 10 OCT images. (A) The results labelled by the physician as the gold standard. (B) The predictions of the improved model.

Following model prediction, contour information is extracted from the resulting plaque images using an edge detection algorithm. The acquired edge data is then overlaid onto the original image, with distinct colors assigned for each plaque type: red for calcified plaques, blue for fibrous plaques, and yellow for lipid plaques. Fig 8 presents the visualization outcomes of 5 OCT images. Fig 8A displays the expert’s annotated results, serving as the gold standard for both training and testing purposes, while Fig 8B showcases the segmentation outcomes achieved by the enhanced model proposed in this paper. Consequently, alongside the segmentation task, the model outputs plaques with localization and labeling information, facilitating rapid and precise visualization and classification. This feature furnishes medical practitioners with a dependable automated tool for the analysis of OCT plaques.

### 3.4 Ablation experiments

To comprehensively demonstrate the impact of incorporating individual modules into the model, ablation experiments were conducted on the calcified plaque dataset. Specifically, the experiments involved the addition of the CBAM and DAC modules, then compared them with the model’s performance prior to enhancement. The results of these experiments are presented in Table 5.

**Table 5:** Ablation experiments.

Method	*precision*	*recall*	*IOU*	*dice*
U-Net	0.926	0.906	0.837	0.905
U-Net + DAC	0.924	0.840	0.902	0.904
U-Net + CBAM	0.921	0.843	0.908	0.907
U-Net + CBAM + DAC	0.922	**0.924**	**0.842**	**0.912**

Incorporating either the CBAM or the DAC module into the original U-Net network has led to marginal enhancements in *dice*, *IOU*, and *recall* metrics. These findings suggest that the integration of these modules yields notable benefits in refining the accuracy of plaque recognition. The *precision* exhibited a decrease when solely incorporating either the CBAM or DAC module, potentially attributable to the model’s heightened tendency to misclassify the background as a plaque in pursuit of capturing more plaque samples. However, when both modules were integrated, the model demonstrated optimal performance.

In order to fully assess the usefulness of the DAC module, the following comparison experiment was performed. Replace all Atrous Convolutions in the model with ordinary convolutions, and also adjust the convolution kernel size and number of layers to ensure that the receptive field is the same as that of the DAC module. The number of parameters and Dice coefficients of the model were counted, as shown in

Table 6. The results show that the DAC module can achieve the same receptive field expansion with fewer parameters, which is in line with its lightweight design goal. The intensive computation of ordinary convolution may fit better in some local details, and thus the Dice is slightly higher. However, DAC performs better in the accuracy-efficiency trade-off and maintains a more stable performance based on the compression of parameter counts by about 1/3.

**Table 6:** Evaluation of the effectiveness of Atrous Convolutional lightweighting.

Model	Total parameters	Model size	Total convolutional layers	Dice
without Atrous Convolution	223,318,733	851.89 MB	39	0.917
Atrous Convolution	63,935,181	243.89 MB	39	0.912

**Fig 9:**
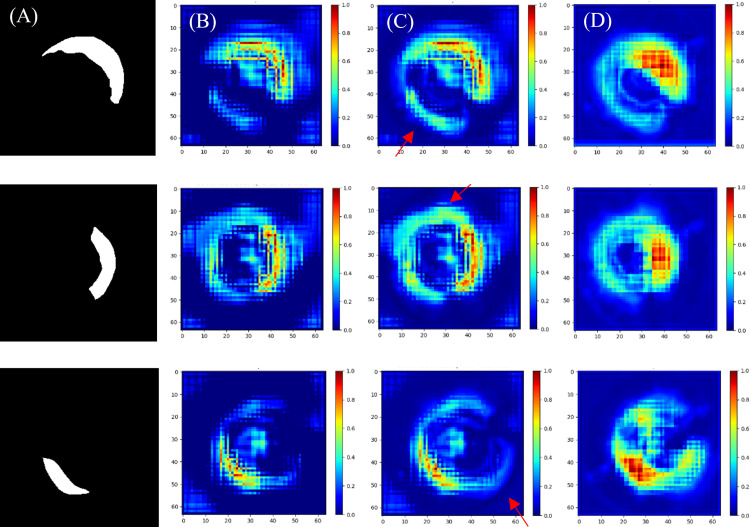
Heatmap comparison. (A) Standard segmented image of the plaque. (B) Original U-Net heatmap. (C) U-Net + DAC heatmap. (D) U-Net + DAC + CBAM heatmap.

To assess the specific contributions of the two modules, DAC and CBAM, heatmap experiments are conducted for feature visualization, as shown in Fig 9. Fig 9A presents the standard segmentation image. Fig 9B illustrates the feature maps after the first up-sampling stage in the original U-Net. Fig 9C and D show the heatmaps of the feature matrices at the same network location, following the addition of the DAC module and the integration of both the DAC and CBAM modules, respectively. The heatmap employs a continuous color spectrum to indicate the distribution of attention intensity, where the red regions correspond to the areas of highest attention and the blue regions denote the lowest response. In Fig 9C, the response region is expanded (as indicated by the red arrow), demonstrating enhanced coverage compared to the base U-Net. This observation validates that the connectivity established by the DAC module through multilevel Atrous Convolution effectively addresses the limitations of the traditional U-Net in extracting global features. The red region in the Fig 9D of the heatmap is both expanded and more evenly distributed compared to when the DAC module is used alone. This suggests that the model’s receptive field is significantly enlarged with the integration of both the CBAM and DAC modules, allowing it to capture more global information. The CBAM module enhances the model’s ability to focus on important feature regions through its spatial and channel attention mechanisms, while the DAC module extends the model’s capacity to capture feature relationships over longer distances by expanding the receptive field.

DAC provides global contextual information, allowing the model to comprehend the overall structure of the image and the interrelationships between different regions, while CBAM focuses on local attention, aiding the model in selecting key features and regions with greater precision. When combined, the DAC and CBAM modules complement each other’s strengths and weaknesses, enabling the model to both capture global information and precisely focus on the most relevant regions and features. This synergy optimizes the model’s feature extraction capability and enhances the final segmentation accuracy. Consequently, the results of the ablation experiments demonstrate that the model performs better when both modules are integrated, as compared to the addition of only one module.

## 4. Discussion

### 4.1 Analysis of results

The algorithm presented in this paper integrates the DAC module and the Attention Mechanism module. It undergoes training on three distinct datasets encompassing calcified, fibrous, and lipid plaques, yielding three prediction models tailored to identify specific plaque types. Upon inputting an OCT image containing multiple plaques, the models selectively output designated plaque types while treating other plaque types as background. This approach enables the classification output of specified plaques within the OCT image. The evaluation metrics unequivocally demonstrate that the enhanced model surpasses not only the original U-Net network but also other comparative networks. This substantiates the efficacy of the proposed series of enhancements for the coronary OCT plaque segmentation task.

**Fig 10:**
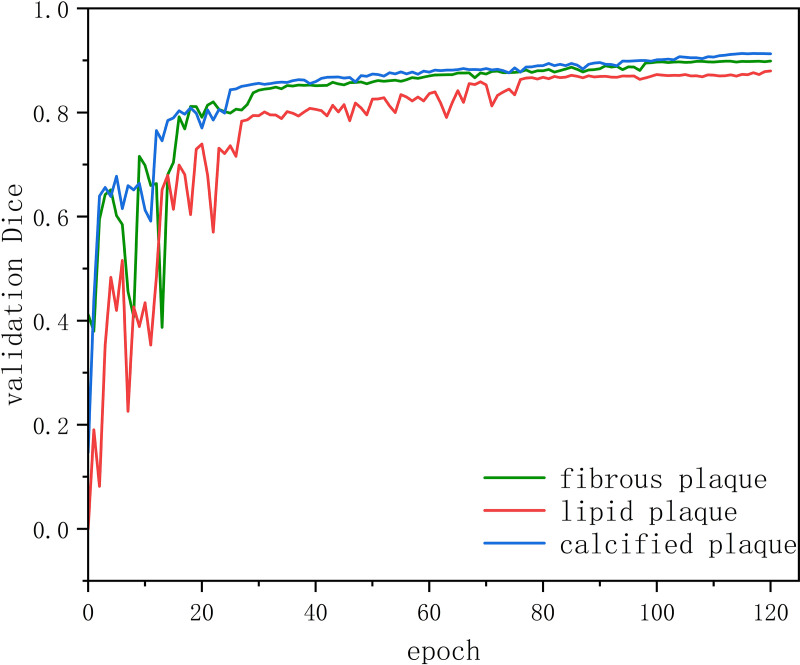
*Dice* coefficient profiles of fibrous, lipid, and calcified plaques on test dataset.

Fig 10 depicts a comparison of the *dice* curves for three distinct plaque types generated by the model proposed in this paper. A value closer to 1 indicates a greater resemblance between the segmentation results and the ground truth. By analyzing the trends and relative positions of these curves, the performance of the model in segmenting different plaque types can be assessed and scrutinized. Furthermore, the smoothness of the curves serves as a reflection of the model’s stability. The *dice* curves pertaining to calcified plaques exhibit notable stability, suggesting the models’ capacity to generate relatively precise predictions across diverse and intricate scenarios. Conversely, the prediction models for fibrous and lipid plaques manifest a higher sensitivity to data instability. Specifically, the curves for fibrous plaques display greater volatility initially before gradually stabilizing, while those for lipid plaques demonstrate pronounced volatility until approximately the 80 epochs training iteration before gradually smoothing out. Through comparative analysis of the performance of different plaque types on the *dice* curves, insights into the model’s prediction variability for distinct plaque types can be gleaned, thereby providing guidance for targeted model enhancements.

## 5. Conclusion

This paper introduces an enhanced model for the automatic segmentation of coronary OCT images. The model incorporates a DAC module, facilitating multiple levels of feature extraction. This module effectively enlarges the receptive field without substantially increasing the data volume, thereby enabling the model to glean richer contextual multiscale information. The incorporation of an attention mechanism enhances the model by allowing it to assign varying weights to different features. This augmentation increases the emphasis on segmentation targets while mitigating interference from background noise, thereby enhancing the model’s learning capability. Comparative experimentation indicates that the model proposed in this paper is better suited for the automatic segmentation of coronary artery plaques compared to existing models. In this study, we employ the same model across three distinct datasets. As a future research direction, enhancing the model could involve analyzing variances in *dice* curve performance across the three plaque dataset types. This analysis could significantly bolster the model’s efficacy and accuracy in diverse plaque detection tasks. Additionally, integrating transfer learning methodologies into coronary artery plaque segmentation tasks holds promise for enhancing segmentation performance and alleviating the burden of dataset annotation.

Currently, large-scale, publicly available OCT datasets are limited. Strict regulations governing medical data access, such as patient privacy protection and data-sharing agreements, hinder the availability of sufficient external central data for validation purposes. Furthermore, the standardization and coordination of cross-center data require substantial time, effort, and resources. Consequently, relying on a single dataset for evaluation may impose constraints on the external validity and generalizability of the findings. Building upon existing research, future work should focus on the following avenues to address these challenges:

(1) Advanced data enhancement techniques such as Generative Adversarial Network (GAN)-based data generation are further explored. By training the generator and the discriminator against each other, the generative adversarial network can generate medical image data closer to the real situation while preserving the characteristics of the real data distribution. This method can not only make up for the problem of insufficient labeled data, but also simulate image data acquired by different centers and different devices, thus improving the robustness of the model in the case of uneven data distribution and differences in acquisition devices.
(2) The use of federated learning technique allows different organizations to collaboratively train a shared model without directly exchanging data, while ensuring data privacy. In federated learning, a shared model is first initialized and distributed to each healthcare institution, and each healthcare institution uses its own local data to train the model for a round of local learning. The risk of data leakage is effectively avoided and data privacy is also ensured.
(3) Multi-Center Collaboration Strategy: Moving forward, we intend to establish collaborations with multiple healthcare organizations to expand the patient data pool by incorporating diverse datasets from different regions and populations. This approach aims to further validate the model’s applicability across a wide range of patient demographics, thereby enhancing its generalizability and robustness.
