# Correction: A plaque recognition algorithm for coronary OCT images by Dense Atrous Convolution and attention mechanism

**DOI:** 10.1371/journal.pone.0333287

**Published:** 2025-09-24

**Authors:** The PLOS One Staff

The fifth author, Cheng Zhang, is incorrectly noted as the corresponding author. The correct corresponding author is Ying Zhang. Ying Zhang’s email address is: chzhangying@126.com.

The publisher apologizes for the error.
